# Internal State Dependent Odor Processing and Perception—The Role of Neuromodulation in the Fly Olfactory System

**DOI:** 10.3389/fncel.2018.00011

**Published:** 2018-01-30

**Authors:** Sercan Sayin, Ariane C. Boehm, Johanna M. Kobler, Jean-François De Backer, Ilona C. Grunwald Kadow

**Affiliations:** ^1^Neural Circuits and Metabolism, School of Life Sciences, Technische Universität München, Munich, Germany; ^2^Chemosensory Coding, Max Planck Institute of Neurobiology, Martinsried, Germany

**Keywords:** *Drosophila melanogaster*, neuromodulation, internal state, hunger, reproductive state, sickness, microbiota, olfaction

## Abstract

Animals rely heavily on their sense of olfaction to perform various vital interactions with an ever-in-flux environment. The turbulent and combinatorial nature of air-borne odorant cues demands the employment of various coding strategies, which allow the animal to attune to its internal needs and past or present experiences. Furthermore, these internal needs can be dependent on internal states such as hunger, reproductive state and sickness. Neuromodulation is a key component providing flexibility under such conditions. Understanding the contributions of neuromodulation, such as sensory neuron sensitization and choice bias requires manipulation of neuronal activity on a local and global scale. With *Drosophila's* genetic toolset, these manipulations are feasible and even allow a detailed look on the functional role of classical neuromodulators such as dopamine, octopamine and neuropeptides. The past years unraveled various mechanisms adapting chemosensory processing and perception to internal states such as hunger and reproductive state. However, future research should also investigate the mechanisms underlying other internal states including the modulatory influence of endogenous microbiota on *Drosophila* behavior. Furthermore, sickness induced by pathogenic infection could lead to novel insights as to the neuromodulators of circuits that integrate such a negative postingestive signal within the circuits governing olfactory behavior and learning. The enriched emporium of tools *Drosophila* provides will help to build a concrete picture of the influence of neuromodulation on olfaction and metabolism, adaptive behavior and our overall understanding of how a brain works.

## 1. Introduction

Some odors elicit fast, almost reflexive behaviors such as fear and escape, others attract an animal already at the very first time it perceives them. Arguably, there might be behaviors that are appropriate at any life stage and in every situation and are therefore hard-wired into the nervous system. The large majority of behaviors, however, including innate odor reactions do make sense at one time, but should be suppressed at others. Or in other words, they strongly depend on an animal's internal state, its current goals and sensory surroundings. These internal states comprise sleep. Sleep, a so-called global state, is essential in most animals (Lee and Dan, 2012). It affects all brain areas and conceivably most other organs in one way or another (Albrecht, 2012). Other internal states might be less exclusive, but probably similarly global. Here, we review recent works in *Drosophila* olfaction research on three important behavioral and internal states: hunger, reproductive state, and the state of sickness or better, the state of an activated immune response. All these states share that they start in one or few organs of the body, and slowly or rapidly, for a short or longer time, affect the rest of the body and in particular its nervous system.

Being able to smell and recognize odors as specific environmental signals is important to humans and absolutely essential for many other animals including *Drosophila melanogaster* (Ashburner et al., 1986). Odors signal food, danger or mating partners without direct contact to their source. Some odors are initially meaningless and remain so unless experienced with a salient cue or object, but some, often species-specific odors elicit a behavioral response such as appetite or repulsion. Nevertheless, how naïve and experienced animals perceive a given odorant depends on their internal state (Leinwand and Chalasani, 2011). For instance, food odors smell better when we are hungry (Rolls, 2006). Male pheromones are only of interest to the ovulating female mouse (Dey et al., 2015). *Drosophila* not only shares with humans and other mammals that odor valence depends on context, it also processes odors with an olfactory system that is highly conserved among different species (Bargmann, 2006). Different studies in the fly over the last decade have greatly improved our understanding of how odors are processed, perceived, categorized and learned (Masse et al., 2009; Wilson, 2013; Sachse and Beshel, 2016). Nevertheless, how flexibility and the ability to adapt to a particular behavioral or internal state is built into the olfactory system of any animal remains poorly understood at the molecular, neuronal and circuit levels (Bargmann, 2012; Taghert and Nitabach, 2012; Bargmann and Marder, 2013). While many neuromodulators have been long identified, a causal relationship between a particular neuromodulator or a group of modulators, their neuronal targets in a neural circuit, and the animal's behavior was established only for few reported cases (see below). Therefore, we focus in the coming paragraphs on the role and possibilities of *Drosophila* neuroscience in providing these causal links between the neuromodulator(s), a neural circuit, and behavior.

### 1.1. The *Drosophila* olfactory system

As mentioned above, the *Drosophila* olfactory system resembles in many ways the mammalian olfactory system (Vosshall and Stocker, 2007) Figure 1. Peripheral olfactory receptor neurons (ORNs) located in hair-like structures, the so-called sensilla, on two of the fly's external appendages, the third segment of the antenna and the maxillary palp, detect the airborne cue via specific receptor molecules. Insects possess three classes of olfactory receptors, the olfactory receptors (ORs) (Vosshall et al., 2000), the gustatory receptors (GRs) (Jones et al., 2007; Kwon et al., 2007), and the ionotropic receptors (IRs) (Benton et al., 2009). While ORs and GRs are, like their mammalian counterpart, seven transmembrane receptors, IRs are related to glutamate receptors and share their structure of ion channels (Abuin et al., 2011). In contrast to the mammalian seven transmembrane receptors, ORs and GRs function as (primarily or exclusively) ion channels rather than as classical G-protein coupled receptors (Sato et al., 2008; Wicher et al., 2008). Nevertheless, similar to mammals, each ORN expresses usually only one ligand-specific receptor and therefore is tuned to few types of odors (Vosshall et al., 2000). ORs always require another OR, the so-called olfactory receptor co-receptor or ORCO, to function (Benton et al., 2006). Similarly, most IRs also appear to function as heteromers with another co-IR (Abuin et al., 2011).

**Figure 1:**
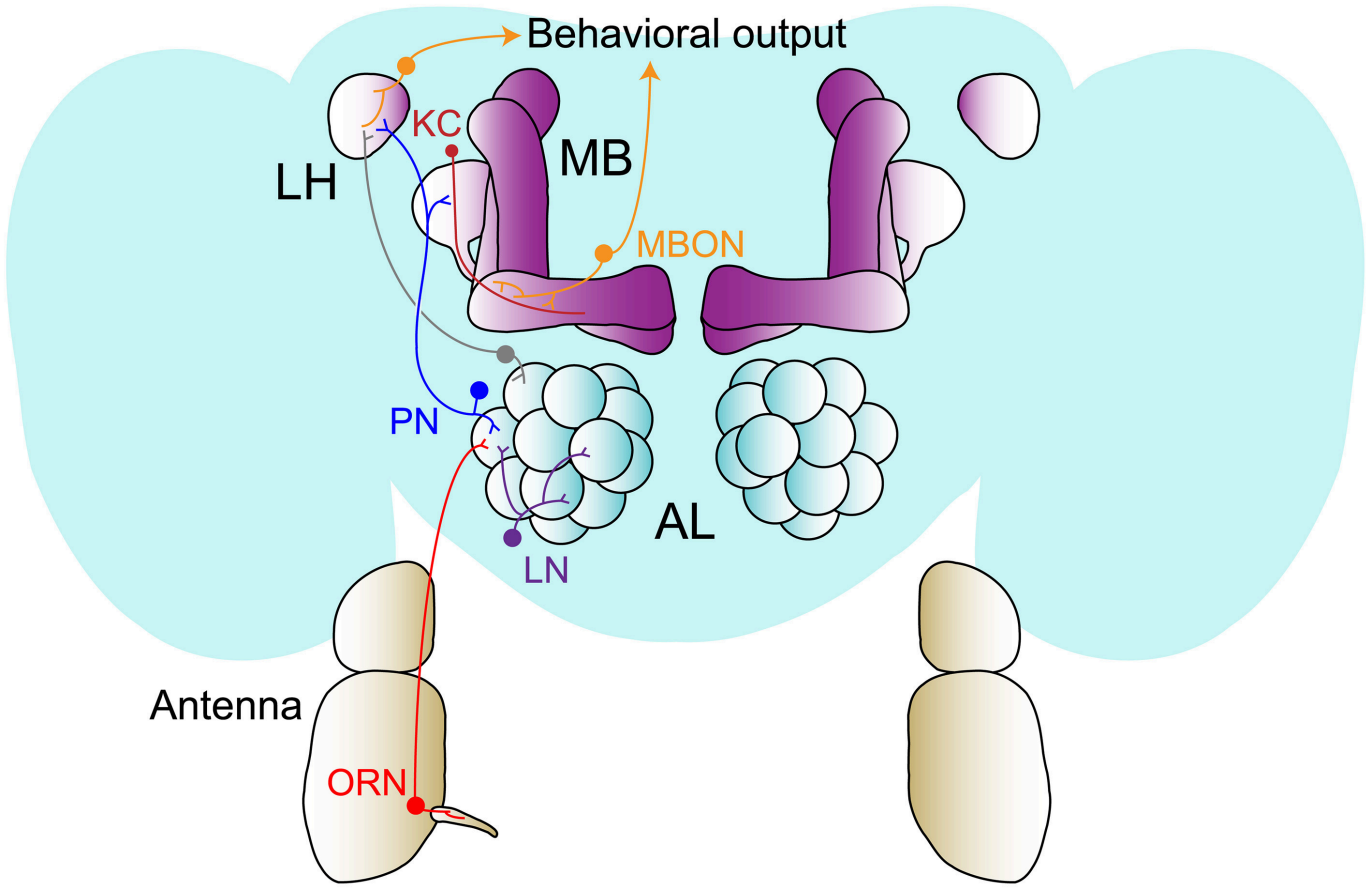
Organization of the olfactory system in *Drosophila melanogaster*. Odors are detected by olfactory receptor neurons (ORN) located in the sensilla of the antennae (and the maxillary palp, not shown). They send axons to specific glomeruli in the antennal lobe (AL) where they form synaptic contacts with projections neurons (PN) and local interneurons (LN). PNs project to higher brain centers, the mushroom body (MB) and/or the lateral horn (LH). In the MB, PNs form en-passant synapses with the Kenyon cells (KC) that convey olfactory information to MB output neurons (MBONs).

ORNs expressing the same receptor or receptor pair send their axons from the peripheral sensilla through a common nerve bundle into the brain, where they innervate glomerular structures in the antennal lobe (AL), the equivalent to the olfactory bulb, in a receptor-type specific manner. Optogenetic activation of one distinct glomerulus is in some cases sufficient to replace an odor in eliciting an attractive or aversive behavioral response [e.g., CO_2_ can be replaced by optogenetic activation of the V-glomerulus (Suh et al., 2007)]. More frequently, however, odors and natural odor blends bind and activate multiple receptors and glomeruli, and only the combined activation of all glomeruli represents the complete perception of a smell. These glomerular activation patterns are further shaped by local interneurons (LNs), which in the fly can be inhibitory and excitatory, to presumably strengthen or weaken similarities and concentration-dependent effects (Wilson, 2013).

In the antennal lobe, projection neurons (PNs) receive this processed information from the ORNs and pass it on to two higher brain centers, the mushroom body (MB) and the lateral horn (LH) Figure 1. The mushroom body is essential for learning, storing, and re-calling odor associations (Aso et al., 2014b), but more recent work has also implicated it in the modulation of innate odor responses (Bräcker et al., 2013; Cohn et al., 2015; Lewis et al., 2015; Owald et al., 2015). While beautiful anatomical and physiological data suggests an important role for the LH in innate odor valuation (Jefferis et al., 2007), very few studies provide compelling behavioral evidence for this role up to now (Strutz et al., 2014). The mushroom body consists of cholinergic Kenyon cells (KCs) that receive sparse and primarily random odor input from PNs, and provide synaptic output to cholinergic, GABAergic and glutamatergic MB output neurons, the so-called MBONs (Aso et al., 2014a). The relative activity of these MBONs, which is highly plastic, is thought to control state- and experience-dependent behavioral output (Aso et al., 2014b; Hige et al., 2015b). Dopaminergic neurons (DANs) that are situated in two primary clusters in the fly brain (PAM, protocerebral anterior medial and PPL1, protocerebral posterior lateral) govern this synaptic plasticity between KCs and MBONs by responding to and integrating of internal and external sensory cues (Owald and Waddell, 2015). At this point, we know most about their role as teaching signals during associative appetitive and aversive memory formation (see for instance Yamagata et al., 2015). Nevertheless, they do modulate behavior instantaneously (Lewis et al., 2015), and potentially play a much greater role in internal state-dependent olfactory processing and behavior as previously thought (Krashes et al., 2009; Siju et al., 2014; Cohn et al., 2015). Finally, to date little is known about the neurons downstream of MBONs and upstream of DANs.

Thanks to great community efforts, we are beginning to appreciate the complexity of the neural connections within the MB circuit (Eichler et al., 2017; Takemura et al., 2017a), the AL (Berck et al., 2016), and other areas of the fly's nervous system (Takemura et al., 2017b). How this complex connectome interacts with a presumably equally complex network of the around 100 neuromodulators present in the fly is a fascinating question without a conclusive answer. The olfactory system of the fly, nevertheless, is a powerful model. It offers many genetic tools, a connectome and a selection of odor-dependent behaviors, which are easy to assess and score. This can tackle the complexity and provide important insights and pointers for research in higher animals. Several fundamental principles beyond the mere architecture of the olfactory system seem likewise conserved. For instance, hunger states and hormonal changes modulate early olfactory sensory processing in worms, flies, mice, and likely in humans (Root et al., 2011; Jang et al., 2012; Palouzier-Paulignan et al., 2012; Dey et al., 2015; Hussain et al., 2016a). Similarly, the mammalian olfactory bulb or its functional equivalent, the antennal lobe in insects, contain a large number of neurons expressing neuromodulators or their receptors (Carlsson et al., 2010; Giessel and Datta, 2014; Linster and Cleland, 2016). How the behavioral role and circuit mechanisms of higher brain centers such as the amygdala and piriform cortex relate to the insect mushroom body and lateral horn is one of the exciting questions that remain to be fully elucidated.

## 2. Modulation happens at many sites

When observing an animal such as *D. melanogaster*, one can notice different facets of its behavior. The disruption of specific genes or a group of genes can change these behaviors and thereby indicates the importance of particular gene networks. Among such genes are genes encoding for neuromodulators, e.g., neuropeptides, enzymes for the generation of monoamines and other types of neurotransmitters.

Neuromodulators can act as control systems such as open and closed loops and feed-forward or feed-back motifs. Furthermore, neuromodulation can happen at many sites within a particular neural network. In the olfactory system, ORNs, secondary PNs, inhibitory neurons, and different types of neurons in the higher brain centers can be targets of modulation. Likewise, this modulation can concentrate on the pre-synaptic/axonal or post-synaptic/dendritic part of a neuron. The effects range from modification of synaptic strength, i.e., inhibition or facilitation, to changes in intrinsic properties, i.e., altering membrane potential or components of the synapse. By doing so, distinct modulators can have independent effects and can rearrange the network into functional units and subcircuits (Marder and Thirumalai, 2002).

In *D. melanogaster*, a large body of work has been published over the last years with respect to neuromodulators and their impact on behavior. One of the most prominent examples is the role of dopaminergic neurons in the MB. These neurons play a key role in olfactory learning and memory and exemplify the importance of neuromodulation in these processes (Berry et al., 2012; Aso et al., 2014a; Hige et al., 2015a; Owald et al., 2015; Aso and Rubin, 2016; Felsenberg et al., 2017; Hattori et al., 2017; Kaun and Rothenfluh, 2017). While DANs are influencing the pre-synaptic efficacy of synapses between KCs and their output neurons, KCs also synapse directly onto DANs, and DANs synapse directly on the output neurons (Takemura et al., 2017a). This means that within the DAN network, information is processed in parallel and in conjunction, with unaccounted opportunities for feed-back and feed-forward loops resulting in multiple layers of neuromodulation. On top of dopamine, octopamine has been shown to govern olfactory associative memory, as it can affect these dopaminergic circuits via dorsal anterior lateral neurons (Burke et al., 2012; Chen et al., 2012; Wu et al., 2013; Guven-Ozkan and Davis, 2014). A contextual cue or internal state allows a direct effect on the modulatory neurons, such as DANs (Cohn et al., 2015; Lewis et al., 2015; Musso et al., 2015). How different modulators like octopamine and dopamine act together to tune a nervous system to a specific state is not well understood.

An important effort in *Drosophila* neuroscience is to map these neural circuits using high-resolution electron microscopy (EM) and data reconstruction. In the *Drosophila* larva, the dense connectome of the MB was recently published. It unraveled expected and unexpected circuit loops and motifs, such as lateral inhibition, feed-back and feed-across circuits (Eichler et al., 2017). The dense interconnectivity of different modulatory neurons and their circuitry even includes a variety of other areas or cell types in the fly brain, such as astrocytes, with even more neurotransmitters, e.g., serotonin (Huser et al., 2012; Ma et al., 2016; Coates et al., 2017, Zheng et al., in review).

EM circuit reconstruction and other modern tools allow the fly community to target and identify the importance of neuromodulators and their effects on behavior within the framework of a known synaptic network, where downstream and upstream neurons of neuromodulatory neurons can be readily identified. Nevertheless, it does not answer the important question of the biological and ethological role of different neuromodulators and how they convey and orchestrate experience, context, and different internal states such as hunger to guide adaptive behavior and ensure optimal chances of survival and success. We will focus on this question for the rest of the present review and provide examples for different internal states and a variety of neuronal targets of neuromodulation.

## 3. Modulation based on internal state

### 3.1. Modulation in hunger

Metabolic state or hunger are arguably the best studied and understood examples of neuromodulation in *Drosophila* neurobiology. A hungry animal desires food. Hunger governs locomotion, perception, motivational state, and tightly links metabolic conditions and behavior. Starved animals show enhanced locomotor activity due to increased aminergic signaling in the fly central and peripheral nervous system (Yang et al., 2015; Yu et al., 2016). However, flies do not solely rely on this hyperactive locomotion to simply increase the likelihood of encountering food. Instead, flies like other animals use olfaction as a proxy, long-distance cue to identify palatable food patches. Therefore, it is no surprise that neuromodulatory effects allow metabolic states to tightly govern the sense of smell. These modulations help the hungry animal to alter its sensory and behavioral thresholds, to filter and sort the spectrum of sensory cues and to integrate innate odor responses with novel food indicators via associative, appetitive learning.

One important mechanism of hunger-dependent regulation of olfaction is the modulation of peripheral sensory neurons, which presumably changes odor valence representation at the level of the AL (Knaden et al., 2012) and in higher brain centers such as the LH (Strutz et al., 2014). Vinegar, as a food cue, activates ORNs and their respective glomeruli, which drive aversive as well as attractive behaviors (Semmelhack and Wang, 2009). The relative level of “push and pull” between low odor concentration driven attraction and high concentration dependent aversion has been shown to be controlled by two parallel modulatory systems at the level of ORNs (Root et al., 2011; Ko et al., 2015). In a paradigm with freely walking flies, starvation decreases the time required to find a food patch. The behavioral increase is accompanied by a rise in signal amplitude in ORNs projecting to three different glomeruli that respond to the lower appetitive concentrations of vinegar; DM1, DM2, and DM4. By contrast, the neuronal activation induced by aversive higher vinegar concentrations in the DM5 glomerulus was reduced in hungry flies (Semmelhack and Wang, 2009). These changes were due to the cohort activity of short neuropeptide F (sNPF) and tachykinin (DTK), respectively (Root et al., 2011; Ko et al., 2015). Removing sNPF via RNA interference (RNAi) or expression of a dominant negative mutant of the sNPF receptor rendered starved fly behavior indistinguishable from satiated flies (Root et al., 2011). Conversely, the induction of fed behavior in starved flies occurred when sNPF signaling was removed from DM1 glomerulus-innervating OR42b neurons. Moreover, removal of sNPF receptor (sNPFR) in secondary order PNs did not alter foraging behavior, suggesting that sNPF functions in an autocrine mechanism (Root et al., 2011). RNAi and overexpression experiments exclusively in OR42b neurons showed that sNPFR was necessary and sufficient for starvation-induced receptivity. What is the link between metabolic state and sNPFR expression in ORNs? mRNA levels of sNPFR were elevated in ORNs after 4 h of starvation, whereas sNPF levels did not change (Root et al., 2011). A parallel mechanism was observed for the modulation of the aversive vinegar channel (Ko et al., 2015). The DM5 glomerulus was found to be under control by Tachykinin (DTK), a player in metabolism, locomotion, aggression and pheromone detection (Winther et al., 2006; Birse et al., 2011; Song et al., 2014; Shankar et al., 2015). Down-regulation of aversive output from the OR85a/DM5 glomerulus was due to increased DTK receptor (DTKR) in the ORNs during food-deprivation (Ko et al., 2015). Starved flies phenocopied fed flies in the absence of DTKR from ORNs. Furthermore, requirement of DTKR was specific to DM5, while loss of DTKR in attraction-mediating OR42b neurons/DM1 had no effect. In contrast to sNPF, Tachykinin was previously reported to be expressed in LNs (Winther and Ignell, 2010). DTK knock-down in LNs mimics DM5 under fed conditions. Importantly, sNPFR and DTKR mRNA levels are under direct control by a common mechanism, insulin. Inducing insulin signaling constitutively abolished odor approach and downregulated sNPFR and DTKR expression from their respective neurons (Root et al., 2011; Ko et al., 2015). Its worthwhile noting that analogous mechanisms have been implicated in mammalian systems (for a review, McIntyre et al., 2017). The firing rate of mitral cells, secondary order neurons equivalent to PNs, were modulated by insulin-mediated inhibition of potassium Kv1.3 channels (Fadool et al., 2000, 2011).

While insulin seems to be the common regulator of some ORNs, do all ORNs respond to the same modulators? An analysis of antennae from starved and fed flies revealed 34 upregulated and 11 downregulated G-protein coupled receptors (Ko et al., 2015). Why there are so many putative modulators and how they contribute to olfactory processing remain open questions. Another study revealed more than 200 genes that are upregulated in the antenna upon starvation, including neuromodulators such as sNPF, allatostatin and CCHamide (Farhan et al., 2013). One of the underlying reasons potentially explaining such a plethora of neuromodulators is that starvation also alters non-food odor and OR independent food odor responses. For instance, cis-vaccenyl acetate (cVA), a pheromone best known for its role in mating, induces attraction in starved flies, even in the absence of potential mates in both sexes during the experiments. This increased behavioral attraction is accompanied by base-line and odor-dependent amplified firing rates. This was equally observed for ethyl acetate encoding ORs, cVa responsive OR67d and ionotropic receptor IR84a that responds to food cue phenylacetaldehyde. Starved mutants of the peptide CCHamide also displayed a decreased attraction to all of these odors (Farhan et al., 2013). Interestingly, sNPF receptor knockdown in ORNs was not effective in reducing ethyl acetate attraction. In another study, a function for SIFamide (SIFa) at the level of projection neurons has been described, adding another neuromodulator involved in hunger-dependent modulation (Martelli et al., 2017). Here, the authors show that activation of SIFamide producing cells does not elicit any changes in activity at the level of peripheral olfactory receptor neurons, but at the level of olfactory projection neurons. This modulation acts through interneurons of the antennal lobe, LNs. Using ethyl acetate as a cue, artificial activation of SIFa neurons transforms a fed fly's odor response from indifference to attraction, mimicking the situation in starved flies. The removal of SIFa from SIFaminergic neurons abolished the differential activation in a specific glomerulus (DM3) between fed and starved animals. Anatomical and physiological data suggests that the neuropeptides hugin and myoinhibitory peptide (MIP, or AllatostatinB) might have opposite effects on SIFa-dependent modulation of appetitive behaviors. While thermogenetic activation of MIP expressing cells weakens SIFaminergic neuronal activity, hugin positive neurons enhance intracellular calcium levels (Martelli et al., 2017). In addition, while activity of sNPF alone did not induce a change in SIFa positive neurons, sNPF was found to be co-expressed with hugin in hugin-positive cells (Martelli et al., 2017). Hugin also plays a role in the Drosophila larvae, where it was recently shown to act as an inhibitory modulator of feeding behavior and as a promoter of locomotion (Melcher and Pankratz, 2005; Schoofs et al., 2014). The observed behavioral differences between larvae and adults may derive from the difference in the developmental/life stage of the animal or other aspects related to behavioral context. Furthermore, heterogeneity in neuromodulatory profiles could further enrich and explain modulatory flexibility of a circuit via concerted action, and might therefore lead to different and context-dependent behavioral outcomes. Neuromodulation of olfaction upon starvation is not restricted to attractive odors, food cues and pheromones. After starvation, behavioral attraction to benzaldehyde, a potent aversive odor for flies, was observed at low odor concentrations, while fed flies still showed odor aversion. In correlation with this behavioral switch, benzaldehyde responsive receptor neurons, which express the receptor OR7a, also showed increased firing rates during odor stimulation in starved animals (Farhan et al., 2013). However, in projection neurons innervating the OR7a-targeted DL5 glomerulus, starvation-dependent modulation was not observed in calcium imaging experiments. Nevertheless, higher benzaldehyde concentrations were used in this study, which might explain the different results (Martelli et al., 2017). Another aversive odor that is present in the context of the fly's preferred food source, overripe and fermenting fruits, is carbon dioxide. Why CO_2_ is aversive is not fully understood, but it is produced by the flies themselves in response to stress or increased metabolic activity (Suh et al., 2004). The release of this odor in the context of food creates a conflict, where CO_2_ aversion must either be overcome during food seeking or its valence must switch from aversive to attractive. Mimicking the context of food, the behavioral response to a mixture of CO_2_ and vinegar was, nevertheless, indistinguishable from a response to CO_2_ alone (Bräcker et al., 2013). Only the additional context of starvation reduced this aversion behavior with the help of the mushroom body (Bräcker et al., 2013; Lewis et al., 2015). In particular, CO_2_ aversion was dependent on a distinct region of the MB lobes, the MB-β'2 lobe region. This region gave output to aversion-driving MBONs, MBON-β'2mp and MBON-β'2mp_bi, which reacted to CO_2_. In the presence of vinegar, however, this CO_2_ response was significantly dampened (Lewis et al., 2015). Although dopamine had been primarily studied in the context of olfactory memory, this study found that certain DANs in the PAM cluster responded to vinegar in a starvation state-dependent manner and inhibited the output of this lobe region (Lewis et al., 2015). Therefore, during starvation, it appears that innate odor responses, too, are under the control of higher brain centers, in particular the MB. Why this modulation does not take place earlier in the circuit, for instance in the ORNs, is not known. It is possible that modulation at the sensory level would be too slow to allow for fast execution of aversive and escape behavior without the context of other odors hinting at the presence of anything else but putative danger. These studies provide evidence that integrated responses of peripheral and higher brains centers are necessary to maximize flexibility and efficacy in behavioral execution.

Motivational thresholds provide an additional mechanism for hunger-dependent olfactory neuromodulation, via the unpaired1 (upd1) - neuropeptide F (NPF) axis (Beshel and Zhong, 2013; Beshel et al., 2017). Three members of the unpaired gene family, fly homologs of the satiety hormone leptin, are expressed in the brain and fat body of the fly and act through JAK-STAT receptor domeless (Tartaglia et al., 1995; Rajan and Perrimon, 2012; Beshel et al., 2017). While fat body-specific downregulation of upd2 leads to decreased body size, reduced upd1 activity selectively in the central nervous system triggers a significant increase in appetitive olfactory behavior in a 4-arm olfactory area assay (Rajan and Perrimon, 2012; Beshel et al., 2017). Lack of upd1 activity also augmented feeding behavior (Beshel et al., 2017). What are the downstream targets of upd1? In an immunohistochemistry experiment, dome was found to colocalize with NPF, the homolog of human NPY (Brown et al., 1999; Beshel et al., 2017). Of the 25 NPF positive neurons in the fly brain, only four have been shown to be essential for olfactory behavior (Beshel and Zhong, 2013). While NPF neurons responded to both food and non-food odors, neuronal activity was increased only for food odors under starvation when compared to the response in the fed state. NPF positive neuron activation levels correlated with the attraction that flies showed toward an odor in behavior. In an olfactory choice arena, given the choice between air and an odor, or two odors to compare, flies accumulated in the quadrant where the odor eliciting higher NPF activity was present. Furthermore, inducing NPF activity artificially was sufficient to facilitate attraction toward non-food odor in a graded fashion (Beshel and Zhong, 2013). When dome expression was targeted specifically to NPY expressing cells, satiated flies showed increased attraction to the food odors (Beshel et al., 2017). Likewise, internal state-dependent differential neuronal activity to the food odors in NPY neurons was abolished with the disruption of upd1/dome signaling. Reduction of upd1 in all neurons in the fly brain and dome knockdown in NPY neurons resulted in higher calcium signaling in fed state, thus mimicking starved condition (Beshel et al., 2017).

Apart from innate odor responses, starvation also modulates appetitive associative learning and memory. Flies are capable of pairing a neutral or aversive odor cue with positive reinforcing stimuli, for example sugar, which provides sweet taste as well as calories (Huetteroth et al., 2015). However, the expression of this memory is suppressed if flies had access to food between odor training and the memory test, suggesting that hunger gates the degree of memory expression and prevents it when the fly does not require food (Krashes et al., 2009). This gating mechanism is provided by NPF. Artificial activation of NPF signaling overrides this suppression and leads to expression of appetitive memory in fed flies. RNAi mediated knock-down of NPF receptor revealed that a subset of dopaminergic PPL1 neurons was critical for this hunger-dependent learning (Krashes et al., 2009). In line with this, these dopaminergic neurons suppressed learning in starved flies when artificially activated. Therefore, hunger and NPF led to disinhibition of mushroom body output, which drives appetitive behavior. A follow up study showed that specific MBONs are modulated through a subset of PPL DANs. PPL1-γ1pedc targets MBON-γ1pedc>α/β (Krashes et al., 2009; Aso et al., 2014a). MBON-γ1pedc>α/β in turn acts as an inter-neuron, selectively inhibiting MBON M4/M6 cluster activity (Aso et al., 2014a; Perisse et al., 2016). This feed-forward inhibition was also found to be hunger regulated with MBON-γ1pedc>α/β showing higher calcium responses to odor in the starved animal. Interestingly, MBON-γ1pedc>α/β and M4/M6 are involved in innate odor aversion (Lewis et al., 2015; Perisse et al., 2016).

**Figure 2:**
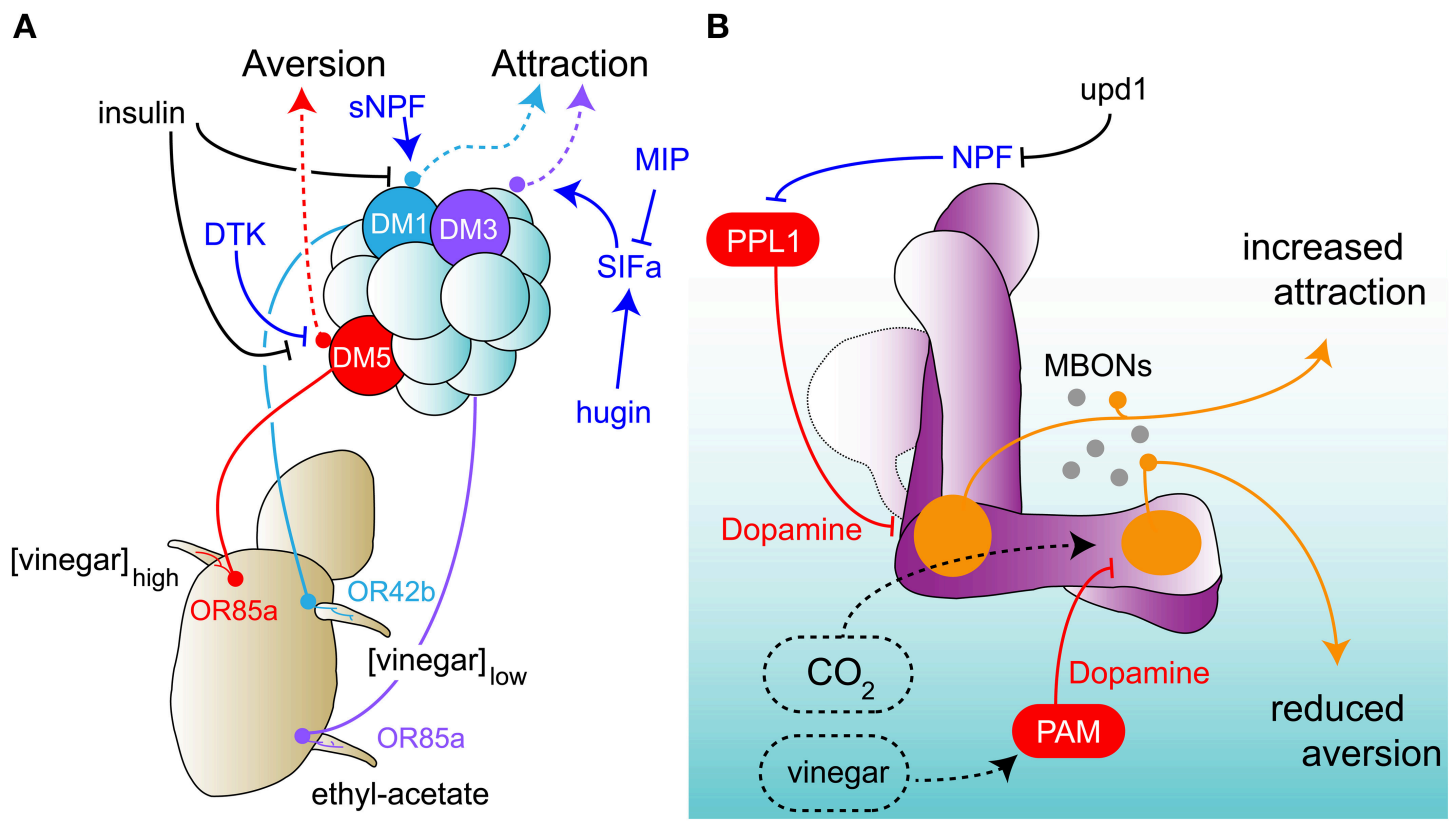
Neuromodulation of olfactory perception in hungry flies. **(A)** Modulation of the peripheral olfactory sensory neurons. Low and high concentrations of vinegar promote attractive and aversive behaviors in *Drosophila*, respectively. In starved flies, the attraction toward low vinegar concentrations is upregulated whereas the repulsive effect of high vinegar concentrations in downregulated. This modulation occurs at the level of ORNs and is mediated by neuropeptides (blue). In fed flies, insulin counteracts these mechanisms by downregulating neuropeptide receptors mRNA. Isoamyl-acetate detection, an other attractive odorant, is upregulated in hungry flies by SIFa, under the control of the neuropeptides MIP and hugin. **(B)** Modulation of olfactory information processing in the mushroom body. In hungry flies, the repulsive effect of CO_2_ is reduced through the activation of PAM dopaminergic neurons by vinegar. Conversely, NPF promotes attraction toward food odors by inhibiting dopaminergic PPL1 neurons. In fed flies, upd1 counteracts this effect. AL, antennal lobe; MB, mushroom body; MBONs, MB output neurons; DTK, tachikinin; NPF, neuropeptide F; sNPF, short NPF; MIP, myoinhibitory peptide; SIFa, SIF amide; PPL1, protocerebral posterior lateral; PAM, protocerebral anterior medial; upd1, unpaired1.

Hunger-induced metabolic changes in the nervous system influence ORN responses and modulate higher brain centers for effective foraging and appetitive learning. Strengthening of attractive channels and inhibition of aversive olfactory pathways therefore appears to occur at several (or every) stage of the olfactory circuitry. Figure 2A recapitulates the neuromodulation on the first level of olfactory processing, the sensory level. The involvement of the MB as the next-higher processing center is summarized in Figure 2B. Why such multi-layered modulation is used is unclear, but it suggests that foraging is under tight control to ensure behavioral expression only when it is in the animal's best interest.

### 3.2. Modulation in reproductive state

For most animals, it is important to master the three components of reproduction: courtship, mating and reproductive success with respect to reproductive fitness. These behaviors are often influenced by neuromodulation and induced downstream of a chemosensory cue such as a pheromone. Courtship and sexuality regulation has previously also been linked to neuromodulators, such as dopamine and octopamine (Certel et al., 2007, 2010; Keleman et al., 2012; Zhou et al., 2012; Rezával et al., 2014; Kuo et al., 2015; Montague and Baker, 2016; Chen et al., 2017; Lim et al., 2017). Mating, however, appears to correlate more often with neurotransmitters engaging glutamate and GABA signaling (Pavlou and Goodwin, 2013; Pavlou et al., 2016; Lim et al., 2017). In the so-called post-mating switch, which includes suppression of re-mating and induction of egg-laying, dopamine, octopamine and certain hormones adapt the sensory perception of females to their reproductive state needs (Ribeiro and Dickson, 2010; Rezával et al., 2012, 2014; Walker et al., 2015; Corrales-Carvajal et al., 2016; Hussain et al., 2016a,b). To ensure reproductive fitness, sensory neurons are in addition modulated by neuropeptides to detect best nutrients and conditions for their offspring. To optimize the action sequence from courtship to mating and finally to offspring fitness, several layers of neuromodulation appear to be necessary to presumably provide sufficient flexibility and stability to the reproductive process of the species.

#### 3.2.1. Modulation during courtship

Courtship behavior in *Drosophila* is based on multiple sensory cues including vision, audition and chemosensation (Greenspan and Ferveur, 2000). Even though vision is a factor, flies can mate in the dark (Payne, 1911; Spieth and Hsu, 1950), indicating that there is more happening than meets the eye. Neuromodulation plays an important role during pheromone and olfactory processing in this behavioral context.

Courtship and subsequently mating requires the detection and recognition of a potential mating partner as a first step. Visual cues such as shape and size are indicators of attractiveness (Agrawal et al., 2014). Odor cues such as pheromone-scented fly dummies are able to modulate the duration of chasing behavior of males significantly, too (Agrawal et al., 2014). This is measured by looking at the important steps within courtship behavior: approach, chasing time and wing extensions.

One may argue that the efficacy of courtship behaviors such as chasing time and the movement of wings can also be age-dependent. Indeed, sexual function of flies has been shown to decrease with age. However, certain dopaminergic neurons of the protocerebral posteriolateral cluster, i.e., PPL2ab, compensate and enhance courtship behavior and therefore presumably also the sexual drive of aged male flies (Kuo et al., 2015). Interestingly, another study has shown that increasing dopamine levels in these PPL neurons can even drive inter-male courtship behavior (Chen et al., 2017) given that visual cues are present.

Not only higher brain areas undergo changes to ensure mating. Also chemosensory cues may guide the way. Cuticular hydrocarbons act as female pheromones. At the sensory neuron level, OR47b has been identified as a key player in the detection of these pheromones (van der Goes van Naters and Carlson, 2007; Dweck et al., 2015; Lin et al., 2016). In older males, the sensitivity of OR47b is augmented via juvenile hormone (Lin et al., 2016). More specifically, the binding partner for juvenile hormone is *Methoprene-tolerant* (Met). If the expression of Met is knocked down in OR47b sensory neurons, a significant reduction in courtship success has been observed. Therefore, older males have an advantage in detecting a female before a young male.

In addition to OR47b, there are further ORs which are able to detect pheromones. While OR47b and OR88a can detect male and female specific pheromones, OR65a and OR67d are specific to male pheromones, i.e., cis-vaccenyl acetate (cVA) (van der Goes van Naters and Carlson, 2007). A deeper analysis of OR67d neurons showed that cVA works in both sexes to inhibit courtship between males and to push courtship in females. This opposing effect is conceivably linked to the GABAergic and cholinergic PNs coming from the dimporphic glomerulus in the AL and leading toward the LH (Kurtovic et al., 2007; Ruta et al., 2010). Since the LH still is a largely uncharacterized area of the adult fly, its potential neuromodulation remains to be investigated.

Another step in successful courtship behavior is the reduction of competition from other males, solved by aggressive behavior. This behavioral switch in displaying either courtship or aggression has been associated with the neuromodulator octopamine (Certel et al., 2007, 2010). Even though a reduction of octopamine triggered enhanced levels of courtship toward other males, an activation of octopaminergic neurons led to the same behavioral outcome. A more detailed analysis needs anatomical precision: In *D. melanogaster*, one gene responsible for gender differentiation is *doublesex*. The development of sex-morphology is *doublesex*-dependent (Rideout et al., 2010). A gene which is often co-expressed with *doublesex* is *fruitless*. When *fruitless* is disrupted in octopaminergic neurons of the subesophageal ganglion (SEZ), male flies display a higher tendency to court other males (Certel et al., 2010). Since the SEZ is the primary gustatory processing center, there is a chance that pheromone detection through GR neurons is the key to this neuromodulation. However, even though GRs, such as GR32a and GR68a (Bray and Amrein, 2003; Miyamoto and Amrein, 2008) have been implicated in courtship success, a connection between GR32a-expressing neurons and the *fruitless*-expressing neurons has not been found (Miyamoto and Amrein, 2008). Therefore, the exact role of octopamine in courtship behavior needs refinement.

Octopaminergic neurons play yet another role within courtship behavior apart from aggression: After mating, females reject males. Hence, it is essential for a male to find a female that has not been mated. As mentioned earlier, females are presenting cVA due to previous mating with another male. These females should now be considered unattractive targets for mating by other males. Olfactory detection of cVA leads to a strong suppression of courtship and mating attempts in males that have previously experienced rejection by an already mated female (Ejima and Griffith, 2007; Ejima et al., 2007). Surprisingly, activation of octopaminergic neurons also induces this effect in virgin males (Zhou et al., 2012), suggesting that octopamine can substitute as a teaching signal during courtship conditioning. In line with this, silencing of octopaminergic neurons during a display of female rejection had a significant effect and males courted irrespectively of the previous rejection (Zhou et al., 2012). Knock-down of the octopamine receptor in the MB (OAMB) also led to a reduction in courtship learning. Furthermore, OAMB-expressing neurons responded to cVA stimulation, thus strengthening a direct role of OAMB.

Dopamine, similar to octopamine, regulates courtship and the males' experience of it. If olfactory detection of cVA is blocked, e.g., OR67d mutant flies, the courtship learning effect vanishes (Keleman et al., 2012). Activation of dopaminergic neurons in males reduces the courtship learning effect. More specifically, *fruitless*-expressing dopaminergic neurons of the class aSP13 are responsible (Keleman et al., 2012). These aSP13 neurons synapse onto the γ lobe of the MB and modulate its output. However, using a screening approach, another study has shown that the γ KCs themselves are not involved in courtship learning, but rather α/β KCs and PAM dopaminergic neurons (Montague and Baker, 2016). Even though *Drosophila* has four dopamine receptors, namely dDA1, DAMB, dD2R, and DopEcR, only one, i.e., dDA1, was identified to modulate courtship in naïve males (Lim et al., 2017). Whereas naïve males without the learning experience court normally, dDA1-mutant naïve males showed a prolonged courtship initiation. An analysis of α/β and γ KCs revealed that restoring dDA1-expressing neurons in these KCs rescues the effect. However, independently expressing dDA1 in either α/β KCs or γ KCs had no rescuing effect. Due to these ambiguous findings, the authors postulate that the neurotransmitters glutamate and GABA, which can be co-transmitters of dopamine in mammals, have a neuromodulatory effect (Lim et al., 2017).

**Figure 3:**
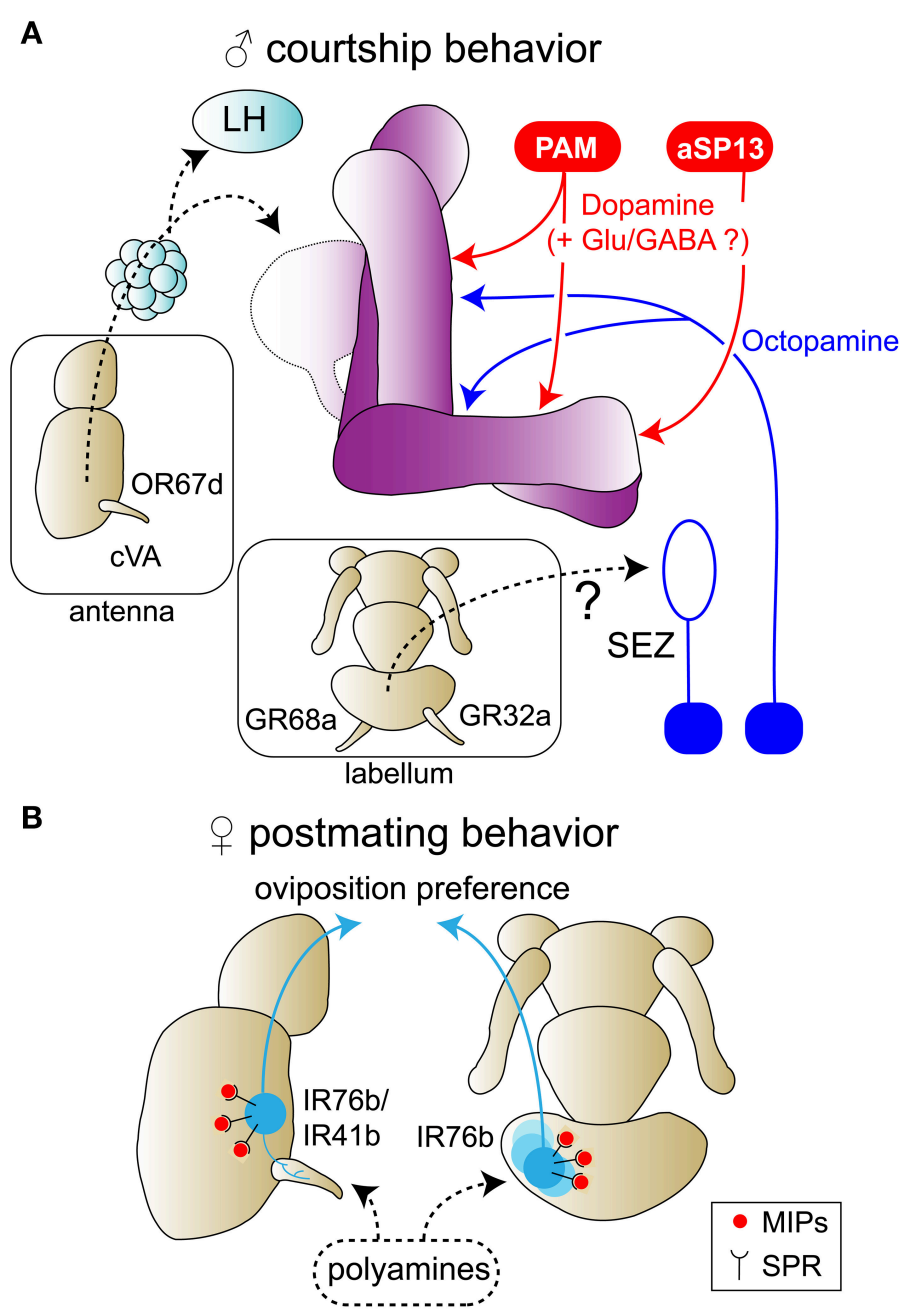
Neuromodulation in reproductive state. **(A)** Modulation of male courtship behavior by pheromones. Pheromones can be detected by olfactory or gustatory receptor neurons in the fly antennas or labellum, respectively. Gustatory neurons are projecting to the subesophageal zone (SEZ) where they might form connections with octopaminergic neurons (blue) known to regulate courtship behavior. The cis-vaccenyl acetate (cVA) pheromone is presented by mated females and inhibits courtship behavior in males that already experienced rejection from an other mated female. This courtship learning is modulated by octopamine in the MB. DANs from PAM and aSP13 clusters (red) also regulate courtship behavior and learning. This might involve co-transmission of glutamate (Glu) or GABA. **(B)** Modulation of female oviposition preference. Mating increases female preference for polyamines. Polyamines detection in both olfactory and gustatory sensory neurons is enhanced through an increase in myoinhibitory peptide (MIP) signaling. This occurs via an upregulation of the expression of its receptor, the sex peptide receptor (SPR), at the membrane of sensory neurons.

In summary, dopamine and octopamine seem to be crucial modulators for courtship behavior. While juvenile hormone may modulate the sensitivity of sensory neurons, a significant amount of modulation happens at higher brain centers. The potential opposing effects of pheromones on males and females may even be linked to the classical neurotransmitters, e.g., GABAergic, glutamatergic, and cholinergic neurons. Figure 3A encapsulates the summary of neuromodulation in courtship behavior.

#### 3.2.2. Mating-induced neuromodulation

As described above, *doublesex* and *fruitless*-expressing neurons play an important role in courtship behavior. From the ORNs which detect pheromones, through different glomeruli in the AL, projections reach higher brain centers: the LH and the MB. To link this with the summary statement of the previous part, it should be mentioned that *doublesex*-expressing neurons are of two types: glutamatergic and GABAergic (Pavlou and Goodwin, 2013; Pavlou et al., 2016). On the one hand, the glutamatergic motor neurons innvervate the genitalia and enable attachment and intromission for the copulation itself. On the other hand, GABAergic inhibitory neurons mediate the uncoupling likely by inhibition of motor neurons. In combination with mechanosensory neurons, which innervate and activate both types, this leads to initiation and end of the copulation process (Pavlou et al., 2016).

Among the known behavioral changes that occur upon mating in *Drosophila* females is a change in their food substrate preferences, presumably due to changed nutritional requirements necessary for egg-production and/or identification of appropriate oviposition sites. Older and more recent work has shown that neuromodulatory mechanism are involved in changing the female's appetite post-mating. For instance, one of the seminal proteins transfered during copulation is sex peptide (SP). If SP is not transferred during copulation, for instance by mating with SP mutant males, female food intake post-mating is reduced compared to their wildtype male-mated peers (Carvalho et al., 2006). Furthermore, mating not only changes the amount, but also increases the female's appetite for yeast and salt again through the transfer of SP and the inhibition of SP receptor expressing neurons in the female's reproductive tract (Ribeiro and Dickson, 2010; Walker et al., 2015; Corrales-Carvajal et al., 2016). Although chemosensory neurons are responsible for the detection of yeast and salt (Walker et al., 2015; Corrales-Carvajal et al., 2016), how and where those neurons are modulated remains unknown.

As mentioned before, *doublesex*-expressing neurons play an important role during mating. These neurons are downstream of SP signaling and have been shown to play an important role in the induction of post-mating behaviors in *Drosophila* females (Rezával et al., 2012). Expression of a membrane-bound form of SP in virgins elicits post-mating behaviors including the display of rejection behaviors toward males and an increase in egg-laying behavior. Furthermore, it is sufficient to inhibit SP receptor (SPR) in *doublesex*-expressing neurons to reduce the post-mating behaviors. In a later paper, the neuromodulator octopamine in *doublesex*-expressing neuron has been shown to modulate post-mating behaviors (Rezával et al., 2014). In virgins, feeding octopamine evoked post-mating responses. In comparison, silencing octopamine in *doublesex*-expressing neurons in mated females revealed a reduction in the post-mating behavior.

The final stage of successful reproduction is reproductive success. In female *Drosophila*, cVA detection may be beneficial for reproductive success, since females may use this olfactory cue to find and mark good oviposition sites (Wertheim et al., 2002). In addition, the consumption of polyamines such as putrescine, spermidine and spermine, can improve reproductive success (Lefèvre et al., 2011). Mated females appear to actively seek out these nutrients (Hussain et al., 2016a,b). Their detection is based on both olfaction and gustation; the ionotropic receptors 76b (IR76b) and IR41a are necessary to detect polyamine odor, while IR76b and the bitter receptor GR66a mediate taste perception (Hussain et al., 2016b). These sensory neurons are modulated at the ORN pre-synapse via endogenously produced MIPs, which are alternative and presumably older ligands of SPR (Hussain et al., 2016a). This peptidergic modulation of peripheral sensory neurons, which appears to be induced by mating, is necessary and sufficient to induce the mated female's increased interest in polyamines. Nevertheless, what triggers the increase of SPR expression in these peripheral neurons initially is not known. It could be a different component of the ejaculate, mating itself or another unknown factor. Finally, this sensory modulation only lasts for a few hours post-mating, while the modulation of female egg-laying behavior and her attraction to higher levels of polyamines are maintained for at least 1 week after mating. It is possible that neuromodulation at other chemosensory processing levels and/or learning play important roles.

This leads to the conclusion that a mixture of allocrine, endogenous and peptidergic modulators are responsible for reproductive success, especially at the different levels of olfactory processing. A recap of the the polyamine detection is depicted in Figure 3B.

### 3.3. State-dependent modulation by sickness and the immune system

Microbial organisms are abundant in the environment and can be found practically everywhere. While a large variety of pathogenic microbes can pose a threat to an animal's survival, many microorganisms also live in close association with animals. This so-called commensal microbiota comprises non-pathogenic microorganisms that reside both in and on an animal's body and play an important role for the host's physiology. Hence, animals must be able to detect microorganisms and distinguish beneficial from potentially harmful or even life-threatening ones when navigating their environment.

*Drosophila melanogaster* feeds on rotten and fermenting fruit, where it is exposed to a variety of different microorganisms including nutritious microbes, which possibly also benefit its microbiota and overall health, as well as pathogenic microbes. Just like hunger or reproductive state govern the animal's motivational state or how it perceives certain chemosensory stimuli, beneficial and harmful microbes and the induced immune response can modify *Drosophila* behavior, too. While much is already known about the composition of the microbiota and its effects on host physiology or the immune processes following pathogenic infection, more recent research is now starting to explore the modulatory influence of the microbiota or pathogens on behavior as well as the underlying neural circuits. Much of this research focuses on the chemosensory senses, and in particular on olfaction, since olfaction constitutes a central mechanism through which *Drosophila* perceives and evaluates its environment and adjusts its behavior in turn.

#### 3.3.1. Modulation via the microbiota

*Drosophila* possesses a relatively simple multispecies microbiota and intestinal structure, thus making it a useful model organism to study host-microbiota interactions and their influences on host behavior. So what does the *Drosophila* microbiota consist of? It mostly comprises yeasts and bacteria from the Enterobacteriaceae and Acetobacteraceae families as well as from the order Lactobacillales (Chandler et al., 2011, 2012; Wong et al., 2011; Broderick and Lemaitre, 2012). The gut bacterial microbiota of natural *Drosophila* populations is very restricted, with laboratory-raised flies exhibiting an even more limited microbiome (Chandler et al., 2011). The acquisition of the microbiota has been proposed to be determined by diet and host physiology (e.g., the pH of the intestine) as well as chance (Chandler et al., 2011). Yet ingestion of exogenous microbiome members, e.g., from decaying fruit, is not only suggested to be a means for the establishment of the *Drosophila* microbiota, but is also required for its maintenance (Broderick and Lemaitre, 2012; Blum et al., 2013).

How does the microbiota interact with its host? The *Drosophila* microbiota can have a variety of effects on its host's physiology, including development, immunity, nutrition, growth and metabolism, epithelium renewal and longevity (e.g., Buchon et al., 2009; Shin et al., 2011; Storelli et al., 2011; Ridley et al., 2012; Combe et al., 2014; Wong et al., 2014; Clark et al., 2015; Li et al., 2016). Interestingly, however, the microbiota can also affect *Drosophila's* behaviors such as nutritional, olfactory and mating preferences as well as oviposition. For example, it has been shown that isogenic *Drosophila* populations raised on either starch or molasses medium develop different microbiota and, when mixed, prefer mating partners reared on the same medium. This preference lasted for 37 generations (Sharon et al., 2010). Antibiotic treatment abolished this medium-induced mating preference, suggesting that fly-associated commensal bacteria are responsible for this effect; a hypothesis that was further corroborated by showing that re-infection of antibiotic-treated flies with either the medium-specific microbiota, a mix of *Lactobacillus* sp. or with *Lactobacillus plantarum* alone, restored mating preferences. As they observed an altered cuticular hydrocarbon composition in the different fly groups, the authors propose that the microbiota influences mating preferences by changing sex pheromone levels (Sharon et al., 2010).

Furthermore, it is known that the microbiota impacts on the nutritional and metabolic phenotype of *Drosophila*. Removal of the resident microbiota for example disturbs energy homeostasis and carbohydrate allocation patterns (Ridley et al., 2012), and the microbiota also affects how nutrients are utilized, e.g., by promoting protein nutrition, modulating lipid/carbohydrate allocation and by provisioning B vitamins (Wong et al., 2014). Thus, in addition or due to the microbiota-host interactions on the physiological level, the microbiota also determines the nutritional preferences of *Drosophila*. In fact, commensal bacteria together with essential amino acids have been posited as central modulators of *Drosophila* food choice (Leitâo-Gonçalves et al., 2017). Flies increased their yeast and amino acid preference as well as their yeast appetite as a reaction to essential amino acid deprivation. Commensal bacteria, specifically the microbiome members *Acetobacter pomorum* and *Lactobacilli*, abolished this increased yeast preference, i.e., the appetite for proteinaceous food. In addition, the microbiota also influences egg-laying behavior: the same study showed that the presence of commensal bacteria similarly rescued the deficits in egg-laying brought about by depriving the flies of essential amino acids.

Another study that elucidates how the microbiota brings about behavioral changes focuses on the consequences of microbe-microbe metabolic exchange on *Drosophila* olfactory and egg-laying behaviors (Fischer et al., 2017). Here, flies preferred a co-culture of two representative microbiome members, i.e., yeast and an acetic acid bacterium, to the same mixture grown separately and combined before testing; a preference mostly mediated by the olfactory receptor OR42b. This divergent response is explained by metabolites that are produced exclusively in microbial communities due to microbial interactions, namely ethanol provided by yeast and converted to acetate by acetic acid bacteria. A second behavior affected by this mechanism is oviposition, as flies similarly preferred to lay their eggs in the co-culture. The emergent metabolites hence serve as an indicator for the presence of a beneficial multispecies microbial community, and by detecting those, *Drosophila* is able to adjust its behavior appropriately.

Furthermore, the gut microbiota can affect its host's chemosensory responses by modulating food preferences and foraging behavior (Wong et al., 2017). It has been demonstrated that *Drosophila* changes its olfactory guided microbial preferences depending on past host-microbe association and the gut microbiota composition. Specifically, microbial preferences in axenic flies were different from those of conventional flies in a foraging assay, and flies reared in monoassociation preferred food seeded with the corresponding bacteria. These diverging preferences for beneficial microbes were shown to be contingent on early-life microbial exposure, since inoculation of eggs was sufficient to alter microbial preferences in freshly emerged larvae. The attraction to these microbiota members was mediated mostly by olfaction. The gut microbes further affected flies' nutritional preferences, as the preference for a balanced diet was abolished in flies offered an imbalanced diet with microbial supplementation, suggesting that *Drosophila* is able to balance nutritional needs with the acquisition of beneficial microbes.

Taken together, apart from its impact on host physiology, it is obvious that the microbiota is also able to modulate a variety of *Drosophila* behaviors, such as oviposition, nutritional or olfactory preferences and foraging. However, so far, not much is known about the mechanisms underlying the formation of these behaviors. Future research will thus have to elucidate how this modulation of behaviors is implemented on the neural circuit level, including for example the necessary communication between gut and brain or the involved neuromodulators.

#### 3.3.2. Modulation due to pathogenic infection and sickness

While it is crucial for *Drosophila* to find suitable food sources that contain beneficial microbes, it similarly has to be able to avoid harmful pathogens, which pose a potential threat to survival. These behavioral strategies that are employed in response to pathogenic microbes to minimize the adverse effects of an infection, such as negative chemotaxis, a reduction in feeding or oviposition in the case of *Drosophila*, can be subsumed under the term ‘behavioral immunity’ (de Roode and Lefèvre, 2012) and provide the animal with a powerful protection mechanism against sickness. Olfaction plays an essential role for these behaviors, as the olfactory system and the associated neural circuits are mainly responsible for the detection and avoidance of harmful stimuli.

Besides pathways which describe how *Drosophila* senses and responds to attractive microbes such as yeast (e.g., Christiaens et al., 2014), one specific olfactory circuit has also been found for the detection of detrimental, pathogenic microbes. Geosmin, a microbial odorant produced by some fungi, bacteria and cyanobacteria, has been shown to specifically activate a single class of sensory neurons that express OR56a and target the DA2 glomerulus in the antennal lobe, where they synapse on projection neurons that are similarly specific for geosmin (Stensmyr et al., 2012). The geosmin circuit hence forms a specific, functionally segregated pathway through the antennal lobe to higher brain centers. Interestingly, it can also modulate and even override innate attraction to potent attractive odors such as vinegar. Activation of the dedicated geosmin circuitry prompts feeding aversion and a reduction in egg-laying; suggesting that geosmin as a powerful indicator of toxic microbes helps *Drosophila* avoid potential sites of infection.

In addition, pathogenic bacteria have recently been shown to manipulate host behavior by increasing the pheromone production of infected flies, thereby attracting healthy flies that are in turn infected themselves and hence further spread the bacteria (Keesey et al., 2017). In particular, flies avoided feeding and egg-laying on a food source containing *Pseudomonas entomophila*, a bacterial strain highly pathogenic for *Drosophila*, but did not respond to the odor of *P. entomophila*. In contrast, *Drosophila* was highly attracted to the odor or the feces of infected flies compared to that of healthy flies; a behavior that was shown to be due to an increase in fatty-acid-derived pheromone release via both immune and insulin signaling pathways upon infection with *P. entomophila*. These findings somewhat parallel the results from Sharon et al. regarding the effects of microbiota on *Drosophila* behavior, as that study similarly proposed a change in sex pheromone levels as the reason for the impact of beneficial microbiome members on *Drosophila* behavior (Sharon et al., 2010). Thus, the ingestion of both beneficial and harmful bacteria might, via immune and metabolic mechanisms, cause alterations in physiology that are in turn detected by conspecifics and provoke behavioral changes.

So far, little is known about the precise mechanisms underlying the behavioral changes prompted by pathogenic infection. More recently, however, it has been demonstrated that an altered egg-laying behavior upon infection in *Drosophila* was due to peptidoglycan sensing by octopaminergic neurons (Kurz et al., 2017). A systemic infection with *E. coli* lead to a reduction in female oviposition that was shown to be elicited by peptidoglycan, a component of the bacterial cell wall that also activates the IMD and Toll innate immunity pathways. Detection of peptidoglycan by the fly induced this decrease in egg-laying via NF-κB pathway activation in octopaminergic neurons, which led to a retention of mature oocytes in the ovaries of infected flies. Additionally, oviposition upon bacterial infection was further modulated by a specific isoform of a peptidoglycan-degrading enzyme that counteracts the reduction in egg-laying to prevent an extreme and thus harmful decrease. This study thus highlights a potential mechanism that allows flies to adapt their egg-laying behavior in response to detrimental environmental conditions, with the modulation of octopaminergic neuron activity playing a central role.

Regarding infection avoidance behavior, some pathogens are detected by their odor such as geosmin (Stensmyr et al., 2012). Nevertheless, not all pathogens smell or are in sufficiently high concentrations in a food to be detected. Hence, it is crucial for an animal to form a memory of the chemosensory perception of food that made it sick in order to be able to avoid it in the future and ensure survival. This acquired avoidance of a particular character of a food such as taste or odor (e.g., of the bacteria) after its pairing with an aversive postingestion effect (i.e., the malaise) is known in vertebrates, but also in invertebrates such as *Caenorhabditis elegans, Drosophila* melanogaster larvae or the honeybee.

*C. elegans* exhibits a range of behaviors in response to pathogenic bacteria; it can for example differentiate between beneficial and harmful bacteria and avoid the latter (Pradel et al., 2007; Schulenburg and Ewbank, 2007; Anyanful et al., 2009; Chang et al., 2011). Interestingly, *C. elegans* can actually learn to avoid pathogens: after exposure to and consumption of pathogenic bacteria, *C. elegans* has been shown to avoid odors from the harmful bacterial strain, while its attraction to odors from familiar non-pathogenic bacteria was increased; a process that required the upregulation of serotonin expression in chemosensory neurons (Zhang et al., 2005). This indicates that the rise in serotonin serves as the negative reinforcing stimulus upon infection with harmful microbes. Interestingly, exposure of *C. elegans* to harmful bacteria in the first larval stage led to long-term aversion of these bacterial odors that was maintained throughout adulthood; and this imprinted aversion was shown to depend on distinct circuits for both formation and retrieval of the imprinted memory (Jin et al., 2016).

**Figure 4:**
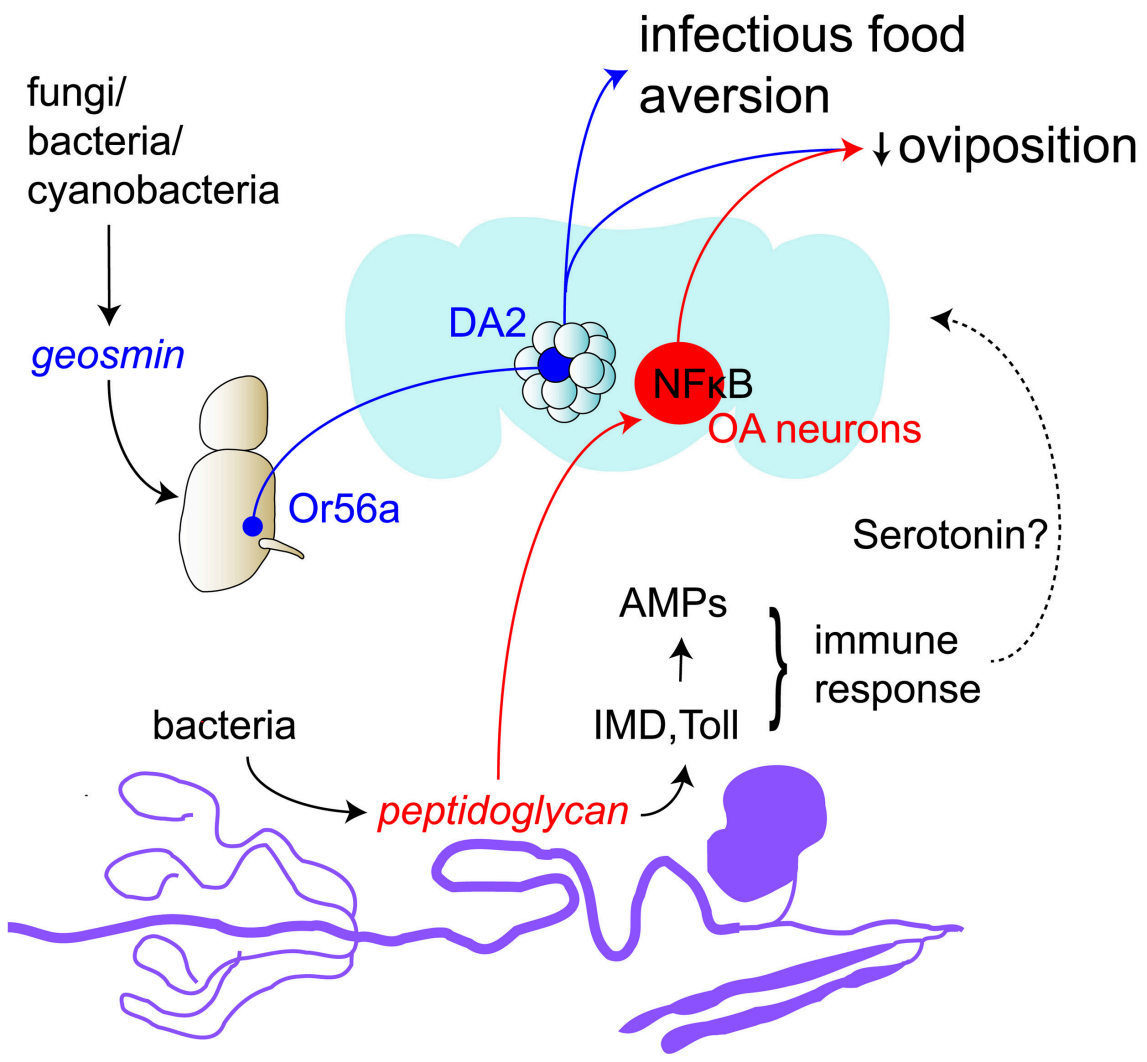
Neuromodulation in sickness and behavioral immunity (blue) Drosophila can avoid feeding or laying eggs on infected food by detecting geosmin produced by some fungi, bacteria and cyanobacteria. Geosmin activates ORNs expressing OR56a that project to the DA2 glomerulus in the antennal lobe. They form synaptic contacts with projection neurons specific for this pathway. (red) In infected flies, octopaminergic neurons (OA neurons) can detect peptidoglycan, a component of the bacterial cell wall, and induce a decrease in egg-laying in infected flies. (black) Peptidoglycan also activates the innate immune response via the IMD and Toll pathways that lead to the production of antimicrobial peptides (AMPs). Studies from *C. elegans* and honeybees suggest that serotonin could form the link between the malaise caused by the ingestion of a pathogen or a toxin and the learned avoidance of the infected food.

Such behaviors have also been shown in insects like the honeybee, which can learn to associate the negative post-ingestive consequences of toxins with the taste of those toxins and the odor present at feeding (Wright et al., 2010). This paradigm mimics the avoidance behavior upon bacteria-induced malaise and similarly required serotonin, since blocking of serotonin receptors abolished the ability of honeybees to associate the odor with the onset of sickness. In line with the results from *C. elegans*, these findings also point to serotonin as a neuromodulator of the circuits that integrate the negative postingestive signal caused by pathogen infection within the circuits regulating olfactory learning Figure 4.

Research in *Drosophila* has so far not been able to show a similar involvement of serotonin or unveil other underlying mechanisms in more detail; however, there is evidence for learned pathogen avoidance behavior in *Drosophila*. Fruit flies, too, may be able to associate an odor with the intestinal malaise caused by pathogen infection (Babin et al., 2014). Following feeding on a food substrate that was supplemented with an odorant and the highly pathogenic bacterial strain *Pseudomonas entomophila*, flies decreased their attraction to this odorant in comparison to an odor not present during infection. No effect was seen in flies conditioned with a harmless version of the same bacterial strain, suggesting that this behavior was in fact due to bacteria-induced malaise.

*Drosophila* larvae employ a comparable defense strategy in response to pathogenic bacteria: when exposed to a mixture of yeast and *P. entomophila, Drosophila* larvae moved away from the food source; a behavior that was not seen when a harmless mutant version of the strain or the less virulent bacterial strain *Erwinia carotovora carotovora* (Ecc15) were used (Surendran et al., 2017). This evasion behavior was diminished in starved larvae and was shown to be reliant on the release of hugin neuropeptide by hugin neurons, whose activity was decreased upon starvation. This puts hugin forward as a modulator of the larval response to harmful pathogens, with a decrease in hugin making starved larvae more prone to overcome their aversion of potentially detrimental food sources.

Therefore, while there is evidence that *Drosophila* can remember the malaise caused by infection, the detailed mechanisms underlying pathogen avoidance behavior in *Drosophila* and the putative memory formation caused by the corresponding negative postingestive effects remain to be elucidated. Nevertheless, studies from other organisms such as *C. elegans* or the honeybee point to a role of serotonin as a neuromodulator of the involved neural circuits. Future studies in *Drosophila* will hence have to address the interplay between sensory information, physiological change and the neural circuits involved in forming a memory of negative postingestive effects as well as the contribution of neuromodulators such as serotonin.

## 4. Methodology and outlook

Neuromodulation is a testament to the fact that the nervous systems is not a static map (Bargmann, 2012). In order to understand comprehensively how the nervous system is rerouted under modulation, the scientific community needs better tools. These tools can be beneficial for expansive circuit mapping, transgenic access to critical nodes of circuits, monitoring activity over time in neuromodulatory cells and recording the impact of those cells on behavior. Recent developments in *Drosophila* expanded such tools drastically (Venken et al., 2011).

The road map of *Drosophila's* nervous system is about to be completed in larval and adult stages. Of the crucial centers of olfaction, the larval antennal lobe and mushroom body connectome has already revealed unsuspected features of wiring in these centers (Berck et al., 2016; Eichler et al., 2017). The adult mushroom body connectome has also been unraveled (Takemura et al., 2017a). However, without thorough understanding of the connectome's road signs, our vision will be only fractional.

Transcriptomics will be helpful in filling these gaps of knowledge, especially in revealing a particular neurons' arsenal of neuromodulators and receptors over time and different internal states. A recent study published the transcriptome of the 6000 cell *Drosophila* embryo (Karaiskos et al., 2017). Although collecting single-cell subtype neurons is arduous, automated cell collection methods have been utilized previously (Tirouvanziam et al., 2004; Salmand et al., 2011; Berger et al., 2012). For such procedures, the ability to repeatedly and reliably target any cell type is crucial.

The arrival of intersectional genetics, the split-Gal4 system, greatly enhanced the resolution one can achieve with transgenic manipulations (Luan et al., 2006; Pfeiffer et al., 2010; Dolan et al., 2017). The majority of mushroom body output neurons and the innervating dopaminergic neurons are now available as transgenic lines, thanks to the split-Gal4 system (Aso et al., 2014a). The split-Gal4 system also enables elegant physiological and behavioral analyses. Calcium integrators that label neurons with sustained responses over time may reveal differential neuromodulator activity between various internal states (Masuyama et al., 2012; Fosque et al., 2015; Gao et al., 2015).

Activity-dependent and specific immunolabeling of dopaminergic and serotonergic neurons is available (Inagaki et al., 2012; Watanabe et al., 2016). A new version of GRASP (i.e., GFP-Reconstitution Across Synaptic Partners), which is based on reconstitution of split-GFP between pre- and post-synaptic neurons, promises to label synapses dependent on synaptic activity (Macpherson et al., 2015).

In acute monitoring of neuronal activity, advances in imaging techniques provide new opportunities, especially in freely behaving animals. Photo-activatable-GFP (PA-GFP) to track neurons or regions has already successfully been used to for example decipher the pheromone circuit (Ruta et al., 2010). In addition to two-photon imaging in head-fixed flies, transcutical multi-photon imaging and calcium-imaging through cutical windows in freely walking flies allows to directly correlate neuromodulation and behavior (Seelig et al., 2010; Grover et al., 2016; Tao et al., 2017; Zheng et al., 2017).

Large-scale behavioral analyses by themselves are highly valuable, too. For instance, a recent study altered and analyzed more than 2,000 lines that innervate the fly central nervous system in a machine vision based non-supervised fashion (Robie et al., 2017). Ultimately, computational modeling will converge anatomical, behavioral and physiological data to form the basis of our understanding of neuromodulation, from a single protein's three dimensional structure to universal models (Hussain et al., 2016b; Richter and Gjorgjieva, 2017).

*Drosophila melanogaster* with its sixth Nobel prize won recently in 2017 (Morgan, 1933; Muller, 1946; Lewis et al., 1995; Axel and Buck, 2004; Beutler et al., 2011; Hall et al., 2017) shows that this organism remains a highly relevant model organism for research. Maybe the next discovery can unravel fascinating insights into neuromodulation.

## 5. Concluding remarks

In the quotidian environment of any animal, the influence of sensory stimuli is constantly present. Chemosensation and particularly olfaction can play an important role in how the animal perceives this environment. The driver of environmental perception is survival. To survive, an animal need to rely on its internal states. Issues including hunger, reproductive state and sickness are needed to be resolved. Neuromodulators are key to behavioral effects seen under these internal state conditions. Often changes are investigated using behavioral approaches. Hunger has been shown to impact on the sensory level as well as higher brain centers in *D. melanogaster*. In courtship behavior, opposing effects of pheromones may be explained with meticulous modulation. Novel research also includes the effect of the microbiome on changes in behavior. If these changes are based on inner modulation remains to be elucidated. Compared to multiple papers that are out there on olfactory memory, it is moreover worth investigating the effects of negative post-ingestive memories like getting sick. The fly community celebrates its sixth Nobel prize. Let's continue on this and focus on unraveling the multi-layered modulation and its interplay with internal states.

## Author contributions

SS, AB, JK, and IG drafted, wrote and revised the manuscript. J-FD implemented all figures and drafted and revised the manuscript. All authors approved to the final version and its publishing.

### Conflict of interest statement

The authors declare that the research was conducted in the absence of any commercial or financial relationships that could be construed as a potential conflict of interest.
